# Clinical Profiles and Outcomes of Patients Undergoing Endoscopic Retrograde Cholangiopancreatography in a Tertiary Care Center

**DOI:** 10.7759/cureus.55065

**Published:** 2024-02-27

**Authors:** Deni Joseph, Ramu Muraleedharanpillai, Sandesh Kolassery, Saji Sebastian

**Affiliations:** 1 Department of Medical Gastroenterology, Government Medical College Kottayam, Kerala, IND

**Keywords:** biliary tracts endoscopy, git endoscopy, clinical profile of ercp cases, complications of ercp, obstructive jaundice, gastroenterology and endoscopy, endoscopic retrograde cholangiopancreatography (ercp)

## Abstract

Introduction

Endoscopic retrograde cholangiopancreatography (ERCP) is a lifesaving therapeutic procedure that carries its own complexities and risks. Clinicians use ERCP as a therapeutic tool for the treatment of biliary stones, malignant obstruction, and acute cholangitis.

Aim

In our study, we aimed to analyze the clinical profile and outcome of patients who underwent ERCP in the Department of Medical Gastroenterology from August 2021 to February 2023 at Government Medical College Kottayam in India.

Materials and methods

We conducted a retrospective study using data from patients who underwent ERCP in the Department of Medical Gastroenterology from August 2021 to February 2023 at Government Medical College Kottayam. We used a semi-structured questionnaire, pro forma, laboratory investigation reports, and SPSS Statistics software (IBM Corp., Armonk, NY) for our data analysis. We included all patients older than 18 years.

Results

In our study 65% of the patients were female. The primary indication for ERCP was common bile duct stones. Of the 216 attempted ERCP cases, we performed successful cannulation in 201 patients, a success rate of 93%. The cannulation time was less than five minutes in the majority of the cases and more than five minutes in 30% of the cases. The commonest type of ampulla in our study was Type One. In our study patients with chronic obstructive pulmonary disease had an increased risk of developing post-ERCP pancreatitis. The most common complication in our study was pancreatitis, which occurred in 29 cases (14%). Only three cases had moderate to severe pancreatitis requiring a prolonged hospital stay of more than three days. There was one fatality immediately following ERCP probably owing to sepsis-induced myocarditis. Of the 201 cases, 15 (7.5%) required precut sphincterotomy.

Conclusion

Analysis of data from patients who underwent ERCP in our department showed that the procedure is safe and effective in treating biliary disorders. The successful cannulation rate and complication rate with ERCP in our tertiary care center are at par with other published data.

## Introduction

Endoscopic retrograde cholangiopancreatography (ERCP) is a lifesaving therapeutic procedure with complexities and risks. The first ERCP was performed in 1968 by McCune et al. in Japan [1]. Initially, ERCP was used as both a diagnostic and therapeutic tool. In 1973, Dr. Meinhard Classen in Germany and Dr. Keiichi Kawai in Japan simultaneously undertook the first biliary sphincterotomy. However, with technical advancements in imaging modalities such as magnetic resonance cholangiopancreatography (MRCPs), endoscopic ultrasounds, and computed tomography (CT) scans, ERCP is now used solely as a therapeutic tool [2]. ERCP carries various risks, including perforation, pancreatitis, bleeding, and infection. Therefore, the use of ERCP as a diagnostic tool is not recommended because safer effective diagnostic tools are now available.

ERCP is used to treat biliary stones, malignant obstructions, acute cholangitis, malignant and benign biliary strictures, and postoperative biliary injuries. In cholangitis, ERCP and urgent biliary drainage are lifesaving procedures. Early ERCP can reduce 30-day mortality in patients with acute cholangitis. Any delay greater than 48 hours is associated with a disproportionate increase in the length of the hospital stay and additional adverse outcomes, including hypotension. ERCP is typically done with conscious sedation, but in unstable patients, full general anesthesia with endotracheal intubation is used.

ERCP in North India is common because of the area’s increased incidence of gallstones. Gallstone disease is less prevalent in South India than in North India. Therefore, fewer ERCP cases occur in South India. We studied the clinical profiles and outcomes of patients who underwent ERCP in a tertiary care center and training center in South India.

## Materials and methods

We analyzed retrospective data from August 2021 to February 2023 regarding patients undergoing ERCP in an endoscopy suite at Government Medical College Kottayam, Kerala, India. All patients were older than 18 and provided their written informed consent prior to undergoing ERCP. All procedures were performed by or under the guidance of three experienced endoscopists who had performed more than 100 ERCPs.

All ERCP procedures were performed using a side-viewing endoscope (TJF 180, Olympus, Tokyo, Japan). Selective cannulation of the common bile duct (CBD) was done using a triple lumen sphincterotome (ULTRATOME, Boston Scientific, USA) and a 0.025-inch guide wire (Visi Glide Olympus, USA). The outcome of the ERCP procedure was considered successful if biliary drainage could be obtained in all cases with biliary sepsis. The procedure was a failure if biliary drainage could not be done. In other cases, depending upon the indications for the procedure and its outcome, the ERCP was considered to be successful or a failure.

Retrospective data of patients who underwent ERCP in the Department of Medical Gastroenterology for a period of 18 months from August 2021 to February 2023 were collected and analyzed using a semi-structured questionnaire, lab investigation reports, and Proforma. SPSS software (IBM Corp., Armonk, NY) was used for all data analysis. All patients older than 18 years undergoing ERCP in the endoscopy suite at Government Medical College Kottayam were included in the study. Patients with high cardiac and pulmonary risk, pregnant women, and patients younger than 18 years were excluded.

## Results

From August 2021 to February 2023, 216 ERCP cases were attempted in the endoscopy suite at Government Medical College Kottayam and 201 were successful. Desired duct cannulation was attained in 201 cases, attributing to a technical success rate of 93%. Of the 15 failed cases, 14 were malignancies, mainly carcinoma in the pancreas with distorted duodenum and ampulla with malignant infiltration. Of the total participants with successful ERCP, 130 were females, and 71 were males (Table 1).

**Table 1:** Sex of study subjects

SEX	FREQUENCY	PERCENTAGE
Male	71	35.3%
Female	130	64.7%
Total	201	100%

The most common indication for doing ERCP was CBD stones, followed by malignancy and other causes (Table 2). Of the 30 cases of malignancies, 15 were carcinoma in the pancreas, seven were periampullary carcinoma, six were cholangiocarcinoma, and two were carcinoma in the gallbladder. Of the 20 miscellaneous cases, 10 were biliary injury after surgery, six were biliary stricture related to chronic pancreatitis, two were probable benign biliary stricture for stenting and brush cytology, and two were pancreatic stenting for a duct leak.

**Table 2:** Diagnosis CBD: Common bile duct

DIAGNOSIS	FREQUENCY	PERCENTAGE
CBD Stone	151	75.1%
Malignancy	30	14.9%
Others	20	10%
Total	201	100%

The most common type of ampulla encountered in our study was Type 1, followed by an almost equal distribution of the rest (Table 3).

**Table 3:** Types of ampulla

TYPE OF AMPULLA	FREQUENCY	PERCENTAGE
Type 1	130	64.7%
Type 2	20	10%
Type 3	21	10.4%
Type 4	30	14.9%
Total	201	100%

In our study, the most common adverse event was pancreatitis, which occurred in 29 patients (Table 4).

**Table 4:** Post ERCP pancreatitis ERCP: Endoscopic retrograde cholangiopancreatography

POST ERCP PANCREATITIS	FREQUENCY	PERCENTAGE
Present	29	14.4%
Absent	172	85.6%
Total	201	100%

The risk of post ERCP pancreatitis in patients with chronic obstructive pulmonary disease (COPD), type 2 diabetes mellitus (T2 DM), hypertension, coronary artery disease (CAD), and increased age were assessed. Using the Chi-square test, we found that COPD patients have an increased risk of developing post ERCP pancreatitis (Tables 5, 6).

**Table 5:** Post ERCP pancreatitis and COPD ERCP: Endoscopic retrograde cholangiopancreatography, COPD: Chronic obstructive pulmonary disease

		COPD		TOTAL
		Yes	No	
Post ERCP pancreatitis	Yes	18	11	29
	No	12	160	172
TOTAL		30	171	201

**Table 6:** Chi-square test DF: Degrees of freedom

	VALUE	DF	ASYMPTOTIC SIGNIFICANCE (2 TAILED)	EXACT SIGNIFICANCE (2 TAILED)	EXACT SIGNIFICANCE (1 TAILED)
Pearson Chi-square test	59.32	1	.000		
Likelihood ratio	43.87	1	.000		
Fisher's exact				.000	.000
Continuity correction	55.06	1	.000		
LInear-by-linear association	59.02	1	.000		
Valid cases	201				

## Discussion

In our study, 65% of the patients were female. The most common indication for ERCP was CBD stone, constituting 75% of the patients. These findings are consistent with several studies [3-5] because gallstone disease occurs more commonly in females. Additionally, patients aged 40 to 60 years made up the largest portion of our study, which is consistent with the age of occurrence of gallstones. Another reason for the high number of cases of CBD stones in our study is that, prior to ERCP, we do endoscopy in all patients to check for infiltration of duodenum in malignant cases. Those with infiltration, those with poor performance status, and frail patients are sent for percutaneous transhepatic biliary drainage (PTBD) rather than attempting ERCP because an intervention radiology department is available. Therefore, most of the malignant cases end up in PTBD rather than undergoing ERCP. This explains the slightly lower number of malignancy cases in our study.

Of the 216 attempted ERCP cases, successful cannulation could be done in 201 patients, a success rate of 93%. Of the 15 failed cases, 14 were malignancy cases, mostly carcinoma at the head of the pancreas. One case was chronic pancreatitis with biliary stricture. A second attempt at ERCP was not done for most patients because a PTBD facility is available in our hospital [6]. Attaining a cannulation rate of greater than 80 % is generally considered satisfactory because we are a teaching hospital. The cannulation time was less than five minutes in around 70% of cases (Table 7). Type 1 ampulla had the least cannulation time, followed by type 3, type 2, and type 4.

**Table 7:** Cannulation time

CANNULATION TIME	FREQUENCY	PERCENTAGE
Less than 5 minutes	140	69.7%
More than 5 minutes	61	30.3%
Total	201	100

The most common complication in our study was pancreatitis, which occurred in 29 cases (Table 8) [7-9]. Only three cases had moderate to severe pancreatitis requiring a prolonged hospital stay for more than three days [10-12]. All other cases had mild pancreatitis and were discharged within three days. Our study’s slightly high occurrence of pancreatitis can be attributed to performing ERCP in a teaching center with trainees. There were 20 post sphincterotomy bleeds [13], but only 10 cases required endoscopic intervention in the form of adrenaline injection. Only one patient required a blood transfusion. There were no cases of perforation and three cases of stent migration-two biliary stent proximal migration and one case of pancreatic stent migration [14]. There was one case of death immediately following ERCP, likely due to sepsis-induced myocarditis [15].

**Table 8:** Complications of ERCP ERCP: Endoscopic retrograde cholangiopancreatography

COMPLICATIONS	NUMBER	PERCENTAGE (%)
Pancreatitis	29	14%
Bleeding	20	10 %
Bleeding requiring endoscopic intervention	10	5%
Perforation	0	0
Cholangitis	5	2.5%
Stent Migration	3	1.5%
Death	1	0.5%

Of the 201 cases, the common bile duct was the desired duct in 195, and the pancreatic duct (PD) was the desired duct in six. In 40 cases, PD was cannulated (Table 9).

**Table 9:** PD cannulation PD: Pancreatic duct

PD CANNULATION	FREQUENCY	PERCENTAGE
Yes	40	19.9%
No	161	80.1%
Total	201	100%

In 30 cases, prophylactic PD stenting was done to prevent pancreatitis (Table 10).

**Table 10:** PD stenting PD: Pancreatic duct

PD STENTING	FREQUENCY	PERCENTAGE
YES	30	14.9%
NO	171	85.1%
TOTAL	201	100%

Out of the total 201 cases, 15 cases required pre-cut sphincterotomy (Table 11).

**Table 11:** Precut

	YES	NO	TOTAL
Precut done	15	186	201
Percentage	7.5%	92.5%	100%

Study limitations

One limitation of our study was that it was a single-center study. Additionally, it was a retrospective study, but a prospective study would have been more useful.

## Conclusions

Our study demonstrated that, despite its complexity, ERCP can be done in our center with a good technical success rate. The complications of ERCP in our study were on par with other published data. The successful cannulation rate was 93% in our center. The most common complication in our study was pancreatitis, occurring in approximately 14% of cases, but the majority of pancreatitis cases were mild and resolved within 72 hours. The cases with significant bleeding were less than 5% in our study. The mortality rate in our study was 0.5%, which is comparable to the latest studies. In short, ERCP, though a complex lifesaving procedure, was found to be effective with an acceptable success rate and complication rate in our setting. Most of the complications related to ERCP could be tackled by Intervention radiology or surgery backup.
